# Methyl 9-*p*-tolyl-8a,9,9a,10,11,12,13,14a-octa­hydro-8*H*-benzo[*f*]chromeno[3,4-*b*]indolizine-8a-carboxyl­ate

**DOI:** 10.1107/S1600536809049447

**Published:** 2009-11-25

**Authors:** B. Gunasekaran, S. Kathiravan, R. Raghunathan, V. Manivannan

**Affiliations:** a Department of Physics, AMET University, Kanathur, Chennai 603 112, India; b Department of Organic Chemistry, University of Madras, Guindy Campus, Chennai 600 025, India; c Department of Research and Development, PRIST University, Vallam, Thanjavur 613 403, Tamil Nadu, India

## Abstract

In the title compound, C_28_H_29_NO_3_, the fused pyrrolidine and piperidine rings of the octa­hydro­indolizine unit exhibit envelope and chair conformations, respectively. The dihedral angle between the naphthalene ring system and the benzene ring is 40.37 (5)°. The crystal packing is stabilized by weak inter­molecular C—H⋯O inter­actions.

## Related literature

For the biological activity of indolizine derivatives, see: Campagna *et al.* (1990 ▶); Malonne *et al.* (1998 ▶); Medda *et al.* (2003 ▶); Pearson & Guo (2001 ▶); Sonnet *et al.* (2000 ▶). For related structures, see: Gunasekaran *et al.* (2009 ▶); Kamala *et al.* (2009 ▶). For details of ring conformations, see: Cremer & Pople (1975 ▶); Nardelli (1983 ▶).
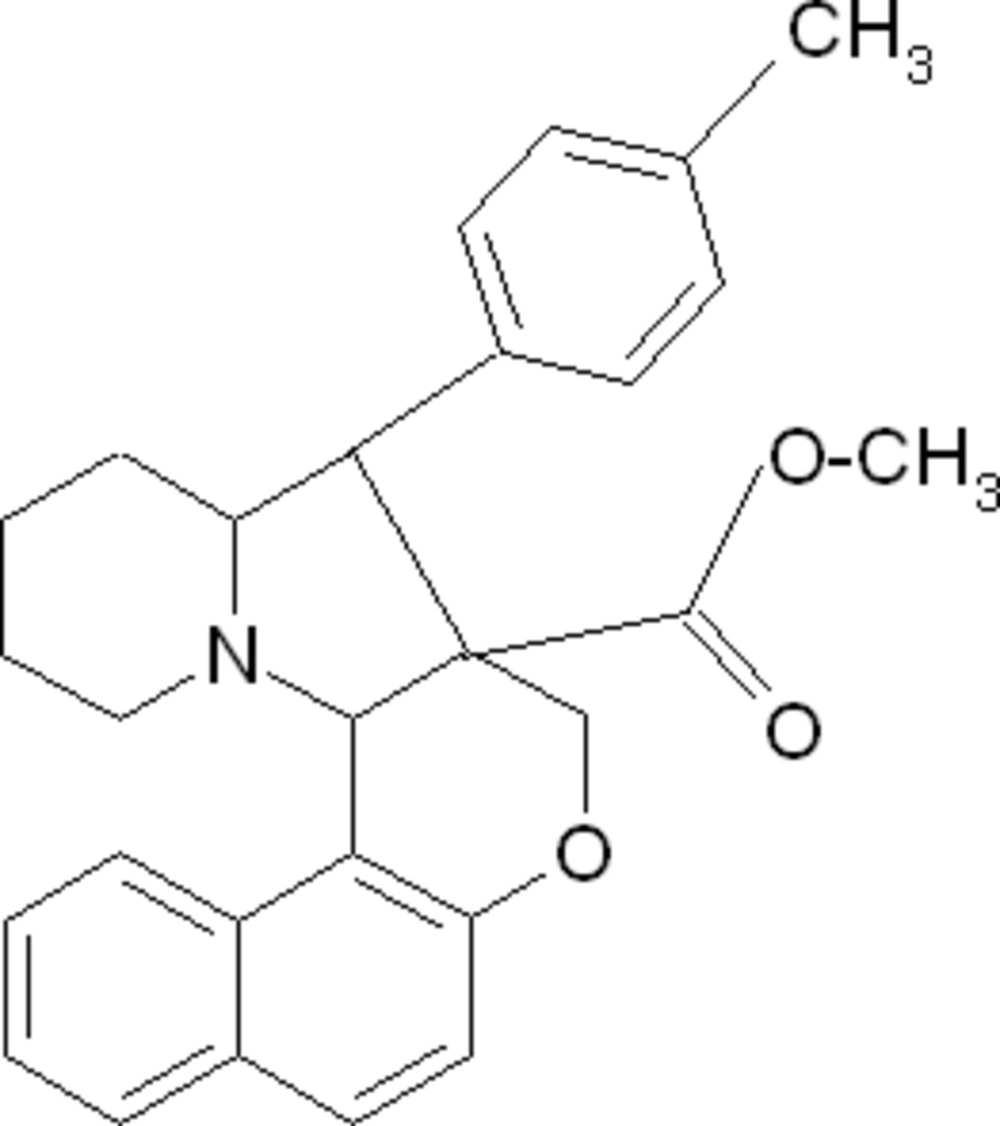


## Experimental

### 

#### Crystal data

C_28_H_29_NO_3_
*M* *_r_* = 427.52
Monoclinic, 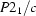

*a* = 11.4842 (9) Å
*b* = 23.0129 (14) Å
*c* = 9.1642 (5) Å
β = 112.725 (2)°
*V* = 2233.9 (3) Å^3^
*Z* = 4
Mo *K*α radiation
μ = 0.08 mm^−1^
*T* = 293 K
0.30 × 0.20 × 0.20 mm

#### Data collection

Bruker Kappa APEXII CCD diffractometer
Absorption correction: multi-scan (**SADABS**; Sheldrick, 1996 ▶) *T*_min_ = 0.976, *T*_max_ = 0.984
27595 measured reflections
5875 independent reflections
3629 reflections with *I* > 2σ(*I*)
*R*_int_ = 0.044

#### Refinement

*R*[*F* ^2^ > 2σ(*F* ^2^)] = 0.051
*wR*(*F* ^2^) = 0.141
*S* = 1.01
5875 reflections
291 parameters
H-atom parameters constrained
Δρ_max_ = 0.22 e Å^−3^
Δρ_min_ = −0.17 e Å^−3^

### 

Data collection: *APEX2* (Bruker, 2004 ▶); cell refinement: *SAINT* (Bruker, 2004 ▶); data reduction: *SAINT*; program(s) used to solve structure: *SHELXS97* (Sheldrick, 2008 ▶); program(s) used to refine structure: *SHELXL97* (Sheldrick, 2008 ▶); molecular graphics: *PLATON* (Spek, 2009 ▶); software used to prepare material for publication: *SHELXL97*.

## Supplementary Material

Crystal structure: contains datablocks global, I. DOI: 10.1107/S1600536809049447/is2492sup1.cif

Structure factors: contains datablocks I. DOI: 10.1107/S1600536809049447/is2492Isup2.hkl

Additional supplementary materials:  crystallographic information; 3D view; checkCIF report

## Figures and Tables

**Table 1:** Hydrogen-bond geometry (Å, °)

*D*—H⋯*A*	*D*—H	H⋯*A*	*D*⋯*A*	*D*—H⋯*A*
C13—H13⋯O2^i^	0.98	2.58	3.496 (2)	156
C7—H7⋯O1^ii^	0.93	2.55	3.198 (2)	127

Symmetry codes: (i) 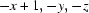; (ii) 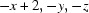.

**Fig. 1.**
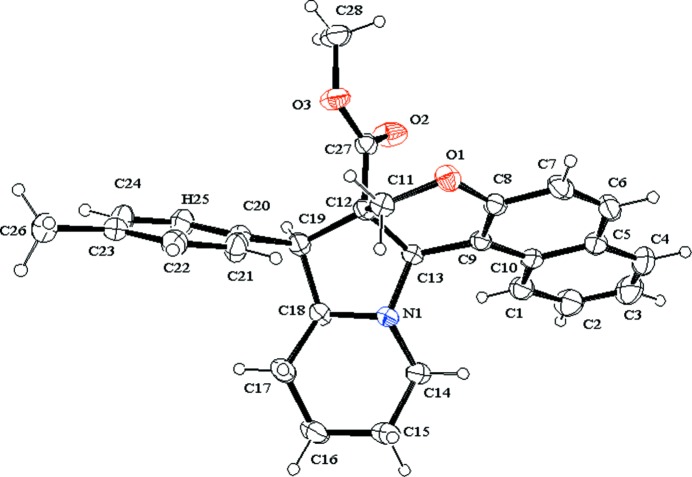
The molecular structure of (I), with atom labels and 30% probability displacement ellipsoids for non-H atoms.

**Fig. 2.**
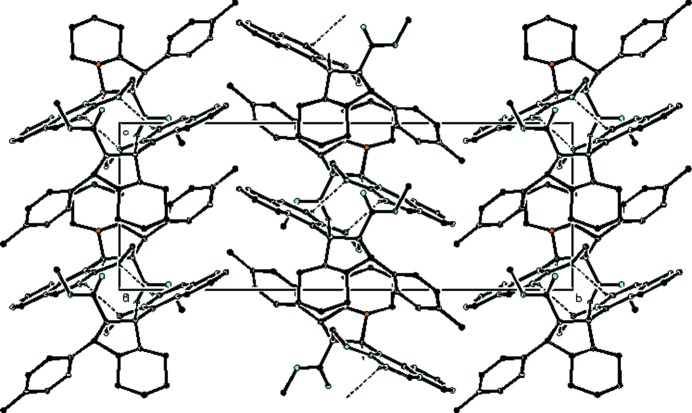
The packing of (I), viewed down the a axis. Hydrogen bonds are shown as dashed lines.

## supplementary crystallographic information

### Comment

The synthesis of biologically active indolizine derivatives continues to attract the attention of organic chemists, because of their wide spectrum of biological activity. Indolizine derivatives have been found to possess a variety of biological activities such as antiinflammatory (Malonne *et al.*, 1998), antiviral (Medda *et al.*, 2003), aromatase inhibitory (Sonnet *et al.*, 2000), analgestic (Campagna *et al.*, 1990) and antitumor (Pearson & Guo, 2001) activities.

The geometric parameters of the title compound (Fig. 1) agree well with reported similar structures (Gunasekaran *et al.*, 2009; Kamala *et al.*, 2009). The mean plane of the naphthalene ring system makes a dihedral angle of 40.37 (5)° with the methyl benzene ring. In the molecule the pyrrolidine ring N1/C13/C12/C19/C18 exhibits an envelope conformation with envelope on C13 with an asymmetry parameter (Nardelli, 1983) ΔC_s_ (C13) = 14.17 (3) and with the puckering parameters (Cremer & Pople, 1975) q2 = 0.4960 (2) Å and φ2 = 205.68 (9)°. The six membered ring N1/C14—C18 exhibits chair conformation with asymmetry parameters ΔC_s_ (N1) = 1.78 (1)/(C16) = 1.78 (1) and with the puckering parameters Q = 0.5855 (2) Å, Θ = 3.86 (3)° and φ = 335.84 (8)°. The sum of bond angles around N1 [333.20 (12)°] indicates *sp^3^* hybridization. The crystal packing is stabilized by weak intermolecular C—H···O interactions.

### Experimental

A mixture of (*Z*)-methyl 2-[(1-formylnaphthalen-2-yloxy)methyl]-3-tolylacrylate (20 mmol) and pipecolinic acid (30 mmol) were refluxed in benzene for 20 h and the solvent was removed under reduced pressure. The crude product was subjected to column chromatography to get the pure product. Chloroform and methanol (1:1) solvent mixture was used for the crystallization under slow evaporation method.

### Refinement

H atoms were positioned geometrically and refined using riding model, with C—H = 0.93 Å and *U*_iso_(H) = 1.2*U*_eq_(C) for aromatic C—H, C—H = 0.98 Å and *U*_iso_(H) = 1.2*U*_eq_(C) for C—H, C—H = 0.97 Å and *U*_iso_(H) = 1.2*U*_eq_(C) for CH_2_, and C—H = 0.96 Å and *U*_iso_(H) = 1.5*U*_eq_(C) for CH_3_.

### Crystal data

**Table:**

C_28_H_29_NO_3_	*F*(000) = 912
*M**_r_* = 427.52	*D*_x_ = 1.271 Mg m^−^^3^
Monoclinic, *P*2_1_/*c*	Mo *K*α radiation, λ = 0.71073 Å
Hall symbol: -P 2ybc	Cell parameters from 4649 reflections
*a* = 11.4842 (9) Å	θ = 2.6–26.3°
*b* = 23.0129 (14) Å	µ = 0.08 mm^−^^1^
*c* = 9.1642 (5) Å	*T* = 293 K
β = 112.725 (2)°	Block, colourless
*V* = 2233.9 (3) Å^3^	0.30 × 0.20 × 0.20 mm
*Z* = 4	

### Data collection

**Table:**

Bruker Kappa APEXII CCD diffractometer	5875 independent reflections
Radiation source: fine-focus sealed tube	3629 reflections with *I* > 2σ(*I*)
graphite	*R*_int_ = 0.044
ω and φ scans	θ_max_ = 28.9°, θ_min_ = 1.8°
Absorption correction: multi-scan (*SADABS*; Sheldrick, 1996)	*h* = −15→15
*T*_min_ = 0.976, *T*_max_ = 0.984	*k* = −31→31
27595 measured reflections	*l* = −12→12

### Refinement

**Table:**

Refinement on *F*^2^	Primary atom site location: structure-invariant direct methods
Least-squares matrix: full	Secondary atom site location: difference Fourier map
*R*[*F*^2^ > 2σ(*F*^2^)] = 0.051	Hydrogen site location: inferred from neighbouring sites
*wR*(*F*^2^) = 0.141	H-atom parameters constrained
*S* = 1.01	*w* = 1/[σ^2^(*F*_o_^2^) + (0.0608*P*)^2^ + 0.3616*P*] where *P* = (*F*_o_^2^ + 2*F*_c_^2^)/3
5875 reflections	(Δ/σ)_max_ = 0.001
291 parameters	Δρ_max_ = 0.22 e Å^−^^3^
0 restraints	Δρ_min_ = −0.17 e Å^−^^3^

### Special details

**Table:**

Geometry. All e.s.d.'s (except the e.s.d. in the dihedral angle between two l.s. planes) are estimated using the full covariance matrix. The cell e.s.d.'s are taken into account individually in the estimation of e.s.d.'s in distances, angles and torsion angles; correlations between e.s.d.'s in cell parameters are only used when they are defined by crystal symmetry. An approximate (isotropic) treatment of cell e.s.d.'s is used for estimating e.s.d.'s involving l.s. planes.

### Fractional atomic coordinates and isotropic or equivalent isotropic displacement parameters (Å^2^)

**Table:**

	*x*	*y*	*z*	*U*_iso_*/*U*_eq_	
C1	0.61198 (18)	−0.14947 (8)	0.0295 (2)	0.0504 (4)	
H1	0.5640	−0.1276	0.0711	0.061*	
C2	0.5662 (2)	−0.20144 (9)	−0.0413 (2)	0.0670 (6)	
H2	0.4879	−0.2144	−0.0469	0.080*	
C3	0.6357 (3)	−0.23540 (9)	−0.1053 (3)	0.0771 (7)	
H3	0.6043	−0.2710	−0.1519	0.093*	
C4	0.7483 (2)	−0.21626 (9)	−0.0990 (2)	0.0676 (6)	
H4	0.7938	−0.2389	−0.1424	0.081*	
C5	0.79886 (19)	−0.16256 (8)	−0.02817 (19)	0.0497 (5)	
C6	0.91374 (19)	−0.14077 (9)	−0.0278 (2)	0.0545 (5)	
H6	0.9598	−0.1630	−0.0714	0.065*	
C7	0.95803 (17)	−0.08848 (8)	0.0344 (2)	0.0490 (4)	
H7	1.0328	−0.0744	0.0307	0.059*	
C8	0.89125 (15)	−0.05504 (7)	0.10510 (17)	0.0383 (4)	
C9	0.78070 (15)	−0.07379 (7)	0.11318 (16)	0.0354 (3)	
C10	0.73006 (16)	−0.12828 (7)	0.04091 (17)	0.0399 (4)	
C11	0.89693 (14)	0.02897 (7)	0.25903 (17)	0.0391 (4)	
H11A	0.9269	0.0688	0.2702	0.047*	
H11B	0.9295	0.0112	0.3631	0.047*	
C12	0.75363 (14)	0.02882 (7)	0.19453 (16)	0.0339 (3)	
C13	0.71155 (14)	−0.03487 (6)	0.18493 (16)	0.0331 (3)	
H13	0.6204	−0.0376	0.1236	0.040*	
C14	0.70537 (18)	−0.10215 (7)	0.39732 (18)	0.0460 (4)	
H14A	0.7485	−0.1326	0.3645	0.055*	
H14B	0.6151	−0.1084	0.3447	0.055*	
C15	0.74380 (19)	−0.10420 (8)	0.57578 (19)	0.0541 (5)	
H15A	0.7177	−0.1410	0.6051	0.065*	
H15B	0.8350	−0.1016	0.6274	0.065*	
C16	0.6842 (2)	−0.05474 (8)	0.6322 (2)	0.0574 (5)	
H16A	0.7163	−0.0549	0.7468	0.069*	
H16B	0.5935	−0.0603	0.5924	0.069*	
C17	0.71307 (19)	0.00345 (8)	0.57545 (18)	0.0509 (5)	
H17A	0.8028	0.0116	0.6254	0.061*	
H17B	0.6679	0.0342	0.6039	0.061*	
C18	0.67275 (16)	0.00141 (7)	0.39653 (17)	0.0387 (4)	
H18	0.5824	−0.0076	0.3506	0.046*	
C19	0.69420 (15)	0.05562 (7)	0.30745 (17)	0.0388 (4)	
H19	0.6096	0.0686	0.2380	0.047*	
C20	0.75513 (17)	0.10776 (7)	0.41025 (17)	0.0418 (4)	
C21	0.88099 (18)	0.11218 (8)	0.51269 (19)	0.0511 (5)	
H21	0.9363	0.0822	0.5162	0.061*	
C22	0.92615 (19)	0.15999 (8)	0.60945 (19)	0.0512 (5)	
H22	1.0115	0.1618	0.6742	0.061*	
C23	0.8483 (2)	0.20499 (7)	0.6126 (2)	0.0521 (5)	
C24	0.7237 (2)	0.20078 (8)	0.5124 (2)	0.0610 (5)	
H24	0.6686	0.2306	0.5113	0.073*	
C25	0.67736 (19)	0.15346 (8)	0.4128 (2)	0.0527 (5)	
H25	0.5924	0.1524	0.3464	0.063*	
C26	0.8973 (3)	0.25562 (9)	0.7251 (2)	0.0732 (7)	
H26A	0.8626	0.2912	0.6709	0.110*	
H26B	0.9877	0.2569	0.7626	0.110*	
H26C	0.8725	0.2509	0.8132	0.110*	
C27	0.69921 (15)	0.05766 (7)	0.03259 (17)	0.0369 (4)	
C28	0.7123 (2)	0.13788 (8)	−0.1198 (2)	0.0593 (5)	
H28A	0.7275	0.1141	−0.1967	0.089*	
H28B	0.7571	0.1739	−0.1082	0.089*	
H28C	0.6235	0.1455	−0.1544	0.089*	
N1	0.73871 (12)	−0.04567 (6)	0.35365 (13)	0.0366 (3)	
O1	0.94386 (11)	−0.00186 (5)	0.15779 (13)	0.0454 (3)	
O2	0.61520 (12)	0.03875 (6)	−0.07961 (14)	0.0632 (4)	
O3	0.75561 (12)	0.10804 (5)	0.02989 (13)	0.0535 (3)	

### Atomic displacement parameters (Å^2^)

**Table:**

	*U*^11^	*U*^22^	*U*^33^	*U*^12^	*U*^13^	*U*^23^
C1	0.0596 (12)	0.0466 (10)	0.0456 (9)	−0.0086 (9)	0.0208 (8)	−0.0026 (8)
C2	0.0793 (15)	0.0566 (12)	0.0630 (12)	−0.0200 (11)	0.0252 (11)	−0.0058 (10)
C3	0.115 (2)	0.0444 (12)	0.0684 (13)	−0.0130 (13)	0.0313 (14)	−0.0120 (10)
C4	0.1003 (18)	0.0460 (12)	0.0592 (11)	0.0105 (12)	0.0339 (12)	−0.0069 (9)
C5	0.0691 (13)	0.0446 (10)	0.0366 (8)	0.0111 (9)	0.0217 (8)	0.0039 (7)
C6	0.0656 (13)	0.0611 (12)	0.0451 (9)	0.0219 (10)	0.0304 (9)	0.0034 (8)
C7	0.0449 (10)	0.0642 (12)	0.0454 (9)	0.0113 (9)	0.0258 (8)	0.0085 (8)
C8	0.0389 (9)	0.0435 (9)	0.0336 (7)	0.0040 (8)	0.0152 (6)	0.0039 (6)
C9	0.0386 (9)	0.0391 (8)	0.0296 (7)	0.0032 (7)	0.0144 (6)	0.0041 (6)
C10	0.0508 (10)	0.0377 (9)	0.0304 (7)	0.0023 (8)	0.0150 (7)	0.0036 (6)
C11	0.0351 (9)	0.0422 (9)	0.0374 (7)	−0.0008 (7)	0.0113 (6)	−0.0001 (7)
C12	0.0320 (8)	0.0387 (8)	0.0292 (6)	0.0017 (7)	0.0099 (6)	0.0004 (6)
C13	0.0324 (8)	0.0383 (8)	0.0292 (6)	0.0000 (7)	0.0125 (6)	0.0015 (6)
C14	0.0553 (11)	0.0462 (10)	0.0397 (8)	−0.0017 (8)	0.0220 (8)	0.0044 (7)
C15	0.0643 (12)	0.0605 (12)	0.0383 (8)	−0.0025 (10)	0.0208 (8)	0.0105 (8)
C16	0.0710 (13)	0.0708 (13)	0.0368 (8)	−0.0016 (11)	0.0278 (9)	0.0028 (8)
C17	0.0609 (12)	0.0597 (12)	0.0347 (8)	0.0025 (9)	0.0212 (8)	−0.0021 (8)
C18	0.0367 (9)	0.0477 (10)	0.0331 (7)	0.0026 (7)	0.0150 (6)	−0.0014 (6)
C19	0.0368 (9)	0.0442 (9)	0.0326 (7)	0.0081 (7)	0.0104 (6)	0.0010 (6)
C20	0.0523 (11)	0.0394 (9)	0.0342 (7)	0.0095 (8)	0.0173 (7)	0.0039 (6)
C21	0.0572 (11)	0.0440 (10)	0.0420 (8)	0.0136 (9)	0.0080 (8)	−0.0043 (8)
C22	0.0644 (12)	0.0451 (10)	0.0382 (8)	0.0018 (9)	0.0134 (8)	−0.0016 (7)
C23	0.0826 (15)	0.0382 (10)	0.0439 (9)	0.0005 (10)	0.0338 (10)	0.0023 (7)
C24	0.0805 (16)	0.0423 (10)	0.0721 (13)	0.0186 (10)	0.0425 (12)	0.0021 (9)
C25	0.0571 (12)	0.0490 (11)	0.0547 (10)	0.0142 (9)	0.0245 (9)	0.0023 (8)
C26	0.121 (2)	0.0436 (11)	0.0669 (12)	−0.0086 (12)	0.0488 (13)	−0.0079 (9)
C27	0.0357 (9)	0.0408 (9)	0.0342 (7)	0.0030 (7)	0.0134 (7)	0.0018 (6)
C28	0.0722 (14)	0.0531 (11)	0.0492 (10)	0.0007 (10)	0.0197 (9)	0.0172 (9)
N1	0.0424 (8)	0.0394 (7)	0.0310 (6)	0.0016 (6)	0.0173 (5)	0.0024 (5)
O1	0.0378 (7)	0.0524 (7)	0.0517 (6)	−0.0038 (6)	0.0234 (5)	−0.0031 (5)
O2	0.0601 (8)	0.0701 (9)	0.0386 (6)	−0.0181 (7)	−0.0037 (6)	0.0108 (6)
O3	0.0660 (9)	0.0462 (7)	0.0395 (6)	−0.0111 (6)	0.0108 (6)	0.0080 (5)

### Geometric parameters (Å, °)

**Table:**

C1—C2	1.365 (3)	C15—H15A	0.9700
C1—C10	1.406 (2)	C15—H15B	0.9700
C1—H1	0.9300	C16—C17	1.519 (2)
C2—C3	1.397 (3)	C16—H16A	0.9700
C2—H2	0.9300	C16—H16B	0.9700
C3—C4	1.346 (3)	C17—C18	1.523 (2)
C3—H3	0.9300	C17—H17A	0.9700
C4—C5	1.412 (3)	C17—H17B	0.9700
C4—H4	0.9300	C18—N1	1.460 (2)
C5—C6	1.410 (3)	C18—C19	1.562 (2)
C5—C10	1.426 (2)	C18—H18	0.9800
C6—C7	1.344 (3)	C19—C20	1.519 (2)
C6—H6	0.9300	C19—H19	0.9800
C7—C8	1.409 (2)	C20—C25	1.386 (2)
C7—H7	0.9300	C20—C21	1.390 (2)
C8—O1	1.3678 (19)	C21—C22	1.382 (2)
C8—C9	1.370 (2)	C21—H21	0.9300
C9—C10	1.432 (2)	C22—C23	1.375 (3)
C9—C13	1.506 (2)	C22—H22	0.9300
C11—O1	1.4286 (18)	C23—C24	1.373 (3)
C11—C12	1.519 (2)	C23—C26	1.513 (3)
C11—H11A	0.9700	C24—C25	1.388 (3)
C11—H11B	0.9700	C24—H24	0.9300
C12—C27	1.522 (2)	C25—H25	0.9300
C12—C13	1.535 (2)	C26—H26A	0.9600
C12—C19	1.569 (2)	C26—H26B	0.9600
C13—N1	1.4755 (17)	C26—H26C	0.9600
C13—H13	0.9800	C27—O2	1.1878 (19)
C14—N1	1.454 (2)	C27—O3	1.3329 (19)
C14—C15	1.521 (2)	C28—O3	1.4404 (19)
C14—H14A	0.9700	C28—H28A	0.9600
C14—H14B	0.9700	C28—H28B	0.9600
C15—C16	1.518 (3)	C28—H28C	0.9600
			
C2—C1—C10	121.47 (19)	C15—C16—H16A	109.4
C2—C1—H1	119.3	C17—C16—H16A	109.4
C10—C1—H1	119.3	C15—C16—H16B	109.4
C1—C2—C3	120.8 (2)	C17—C16—H16B	109.4
C1—C2—H2	119.6	H16A—C16—H16B	108.0
C3—C2—H2	119.6	C16—C17—C18	109.00 (14)
C4—C3—C2	119.6 (2)	C16—C17—H17A	109.9
C4—C3—H3	120.2	C18—C17—H17A	109.9
C2—C3—H3	120.2	C16—C17—H17B	109.9
C3—C4—C5	121.6 (2)	C18—C17—H17B	109.9
C3—C4—H4	119.2	H17A—C17—H17B	108.3
C5—C4—H4	119.2	N1—C18—C17	109.83 (13)
C6—C5—C4	122.05 (18)	N1—C18—C19	104.42 (11)
C6—C5—C10	118.72 (16)	C17—C18—C19	119.63 (14)
C4—C5—C10	119.20 (19)	N1—C18—H18	107.5
C7—C6—C5	121.31 (16)	C17—C18—H18	107.5
C7—C6—H6	119.3	C19—C18—H18	107.5
C5—C6—H6	119.3	C20—C19—C18	115.77 (12)
C6—C7—C8	119.99 (17)	C20—C19—C12	120.39 (14)
C6—C7—H7	120.0	C18—C19—C12	103.14 (12)
C8—C7—H7	120.0	C20—C19—H19	105.4
O1—C8—C9	123.71 (14)	C18—C19—H19	105.4
O1—C8—C7	113.99 (14)	C12—C19—H19	105.4
C9—C8—C7	122.24 (16)	C25—C20—C21	116.22 (16)
C8—C9—C10	118.03 (14)	C25—C20—C19	117.67 (16)
C8—C9—C13	119.49 (14)	C21—C20—C19	125.90 (15)
C10—C9—C13	122.28 (14)	C22—C21—C20	121.74 (17)
C1—C10—C5	117.31 (16)	C22—C21—H21	119.1
C1—C10—C9	123.05 (15)	C20—C21—H21	119.1
C5—C10—C9	119.60 (16)	C23—C22—C21	121.79 (18)
O1—C11—C12	111.72 (12)	C23—C22—H22	119.1
O1—C11—H11A	109.3	C21—C22—H22	119.1
C12—C11—H11A	109.3	C24—C23—C22	116.82 (17)
O1—C11—H11B	109.3	C24—C23—C26	121.77 (19)
C12—C11—H11B	109.3	C22—C23—C26	121.4 (2)
H11A—C11—H11B	107.9	C23—C24—C25	122.03 (18)
C11—C12—C27	110.70 (12)	C23—C24—H24	119.0
C11—C12—C13	107.11 (13)	C25—C24—H24	119.0
C27—C12—C13	111.03 (12)	C20—C25—C24	121.38 (19)
C11—C12—C19	115.12 (12)	C20—C25—H25	119.3
C27—C12—C19	110.62 (12)	C24—C25—H25	119.3
C13—C12—C19	101.87 (12)	C23—C26—H26A	109.5
N1—C13—C9	115.07 (12)	C23—C26—H26B	109.5
N1—C13—C12	99.57 (11)	H26A—C26—H26B	109.5
C9—C13—C12	112.67 (12)	C23—C26—H26C	109.5
N1—C13—H13	109.7	H26A—C26—H26C	109.5
C9—C13—H13	109.7	H26B—C26—H26C	109.5
C12—C13—H13	109.7	O2—C27—O3	122.72 (14)
N1—C14—C15	109.00 (14)	O2—C27—C12	125.07 (15)
N1—C14—H14A	109.9	O3—C27—C12	112.20 (13)
C15—C14—H14A	109.9	O3—C28—H28A	109.5
N1—C14—H14B	109.9	O3—C28—H28B	109.5
C15—C14—H14B	109.9	H28A—C28—H28B	109.5
H14A—C14—H14B	108.3	O3—C28—H28C	109.5
C16—C15—C14	111.17 (15)	H28A—C28—H28C	109.5
C16—C15—H15A	109.4	H28B—C28—H28C	109.5
C14—C15—H15A	109.4	C14—N1—C18	111.72 (12)
C16—C15—H15B	109.4	C14—N1—C13	117.86 (12)
C14—C15—H15B	109.4	C18—N1—C13	103.62 (11)
H15A—C15—H15B	108.0	C8—O1—C11	116.59 (12)
C15—C16—C17	110.99 (14)	C27—O3—C28	116.17 (13)
			
C10—C1—C2—C3	−0.1 (3)	N1—C18—C19—C12	10.03 (14)
C1—C2—C3—C4	−0.9 (3)	C17—C18—C19—C12	133.35 (15)
C2—C3—C4—C5	0.4 (3)	C11—C12—C19—C20	36.45 (19)
C3—C4—C5—C6	−176.85 (19)	C27—C12—C19—C20	−89.96 (16)
C3—C4—C5—C10	1.1 (3)	C13—C12—C19—C20	151.96 (13)
C4—C5—C6—C7	177.23 (17)	C11—C12—C19—C18	−94.42 (15)
C10—C5—C6—C7	−0.8 (3)	C27—C12—C19—C18	139.17 (13)
C5—C6—C7—C8	1.9 (3)	C13—C12—C19—C18	21.08 (14)
C6—C7—C8—O1	−177.48 (15)	C18—C19—C20—C25	−101.78 (17)
C6—C7—C8—C9	−0.1 (2)	C12—C19—C20—C25	133.08 (16)
O1—C8—C9—C10	174.48 (13)	C18—C19—C20—C21	72.7 (2)
C7—C8—C9—C10	−2.6 (2)	C12—C19—C20—C21	−52.4 (2)
O1—C8—C9—C13	−0.6 (2)	C25—C20—C21—C22	−1.1 (3)
C7—C8—C9—C13	−177.70 (13)	C19—C20—C21—C22	−175.66 (16)
C2—C1—C10—C5	1.6 (2)	C20—C21—C22—C23	1.8 (3)
C2—C1—C10—C9	179.52 (16)	C21—C22—C23—C24	−1.3 (3)
C6—C5—C10—C1	175.96 (15)	C21—C22—C23—C26	176.75 (17)
C4—C5—C10—C1	−2.1 (2)	C22—C23—C24—C25	0.2 (3)
C6—C5—C10—C9	−2.0 (2)	C26—C23—C24—C25	−177.77 (17)
C4—C5—C10—C9	179.92 (15)	C21—C20—C25—C24	0.1 (3)
C8—C9—C10—C1	−174.23 (15)	C19—C20—C25—C24	175.10 (16)
C13—C9—C10—C1	0.7 (2)	C23—C24—C25—C20	0.4 (3)
C8—C9—C10—C5	3.6 (2)	C11—C12—C27—O2	133.84 (18)
C13—C9—C10—C5	178.58 (13)	C13—C12—C27—O2	15.0 (2)
O1—C11—C12—C27	−60.21 (17)	C19—C12—C27—O2	−97.33 (19)
O1—C11—C12—C13	60.97 (15)	C11—C12—C27—O3	−47.10 (18)
O1—C11—C12—C19	173.42 (12)	C13—C12—C27—O3	−165.93 (12)
C8—C9—C13—N1	−95.53 (16)	C19—C12—C27—O3	81.74 (16)
C10—C9—C13—N1	89.59 (17)	C15—C14—N1—C18	−61.31 (18)
C8—C9—C13—C12	17.69 (18)	C15—C14—N1—C13	178.87 (14)
C10—C9—C13—C12	−157.18 (13)	C17—C18—N1—C14	63.40 (17)
C11—C12—C13—N1	76.82 (13)	C19—C18—N1—C14	−167.15 (12)
C27—C12—C13—N1	−162.21 (12)	C17—C18—N1—C13	−168.72 (13)
C19—C12—C13—N1	−44.41 (13)	C19—C18—N1—C13	−39.26 (14)
C11—C12—C13—C9	−45.60 (15)	C9—C13—N1—C14	−62.47 (18)
C27—C12—C13—C9	75.37 (16)	C12—C13—N1—C14	176.84 (13)
C19—C12—C13—C9	−166.83 (11)	C9—C13—N1—C18	173.56 (13)
N1—C14—C15—C16	56.0 (2)	C12—C13—N1—C18	52.87 (14)
C14—C15—C16—C17	−53.9 (2)	C9—C8—O1—C11	15.4 (2)
C15—C16—C17—C18	54.2 (2)	C7—C8—O1—C11	−167.33 (13)
C16—C17—C18—N1	−58.24 (18)	C12—C11—O1—C8	−46.71 (18)
C16—C17—C18—C19	−178.89 (15)	O2—C27—O3—C28	−2.3 (2)
N1—C18—C19—C20	−123.56 (14)	C12—C27—O3—C28	178.56 (14)
C17—C18—C19—C20	−0.2 (2)		

### Hydrogen-bond geometry (Å, °)

**Table:**

*D*—H···*A*	*D*—H	H···*A*	*D*···*A*	*D*—H···*A*
C13—H13···O2^i^	0.98	2.58	3.496 (2)	156
C7—H7···O1^ii^	0.93	2.55	3.198 (2)	127

Symmetry codes: (i) −*x*+1, −*y*, −*z*; (ii) −*x*+2, −*y*, −*z*.
